# Cannabis Exposure During Critical Windows of Development: Epigenetic and Molecular Pathways Implicated in Neuropsychiatric Disease

**DOI:** 10.1007/s40572-020-00275-4

**Published:** 2020-05-22

**Authors:** Anna Smith, Farla Kaufman, Martha S. Sandy, Andres Cardenas

**Affiliations:** 1 Division of Environmental Health Sciences, School of Public Health, University of California, Berkeley, CA USA; 2 Center for Computational Biology, University of California, Berkeley, CA USA; 3 Office of Environmental Health Hazard Assessment, California Environmental Protection Agency, Sacramento, CA USA

**Keywords:** Cannabis, Δ^9^-THC, Epigenetics, Prenatal exposure, DOHaD, DNA methylation

## Abstract

**Purpose of Review:**

Cannabis exposure during critical windows of development may have intergenerational physiological consequences disrupting epigenetic programming and marks. This review examines the literature relating to pre-gestational and prenatal cannabinoid exposure and its effect on genes and molecular pathways related to the development of psychiatric disease.

**Recent Findings:**

Developmental cannabis exposure alters epigenetic processes with functional gene consequences. These include potentially heritable alterations in genes and molecular pathways critical for brain development and associated with autism spectrum disorder (ASD), attention deficit hyperactivity disorder (ADHD), schizophrenia, addiction, and other psychiatric diseases.

**Summary:**

Cannabis consumption and mental health illness in adolescents and young adults are increasing in the United States (U.S.), and recent studies suggest that cannabis consumption during critical periods of brain development could contribute to mental health illness through epigenetic mechanisms. These findings warrant future studies and consideration by regulators and health communicators.

## Introduction

Cannabis (marijuana) is the most commonly used illicit psychoactive drug in the United States (U.S.) with an estimated 9.6% of the population aged 12 and older reporting use in the past month [1]. States are increasingly legalizing both recreational and medicinal cannabis use [2]. The majority of new users are under 18 years of age [3], and cannabis use has increased among youth and teens since 2007 [4]. In addition, pregnant women are increasingly using it to mitigate morning sickness. In the U.S., between 2002–2003 and 2016–2017, the adjusted prevalence of past-month cannabis use increased from 3.4 to 7.0% among pregnant women overall and from 5.7 to 12.1% among pregnant women during the first trimester [5]. A recent national survey suggested that the public perception of “great risk” from weekly cannabis use has dropped from 50.4% in 2002 to 33.3% in 2014 [6]. Another recent survey found that 81% of U.S. adults believe that cannabis has at least one health benefit, such as use in pain management, disease treatment, or relief of anxiety, stress, or depression. While 91% of U.S. adults also believe cannabis use has at least one risk, including those associated with legal issues, 9% believe it has no risks, and the American public has an overall favorable view of cannabis [7].

Cannabis is composed of over 400 chemicals, of which over 60 are cannabinoid compounds. The four major compounds include Δ^9^-tetrahydrocannabinol (THC), cannabidiol (CBD), Δ^8^-tetrahydrocannabinol, and cannabinol [8]. The major psychoactive cannabinoid in cannabis, THC, targets the endocannabinoid (eCB) system, which regulates biological processes involved in development and neuroplasticity [9]. It mimics eCB action, exerting most of its effects via cannabinoid receptors (CBR)s 1 and 2. CBR1 is one of the most abundant G protein-coupled receptors in the adult brain, and it is localized in regions important in movement, cognition, attention, emotion, and memory [10]. In animals, expression begins early in the central nervous system (CNS) during embryonic development [11–13]. One study found CBR1 expressed in the human fetal brain at 20 weeks, with high expression in the hippocampus and amygdala [14]. In contrast, CBR2 is mainly expressed in immune cells. Male mitotic germ cells also express a high level of CBR2, whose activation promotes their differentiation and progresses spermatogenesis [15••]. During adolescence, the eCB system continues to facilitate neurodevelopment through its involvement in neuroplasticity and synaptic function. Levels of CBRs fluctuate during adolescence and depend on the brain region. For instance, there is a rapid, sustained increase in CBR binding sites in the striatum that is reduced by half in early adulthood [16], as well as high levels in limbic related regions that gradually decrease by adulthood [17]. Tightly regulated biosynthetic pathways ensure proper signaling throughout development, and correct brain function depends heavily on the temporal and spatial layout of the eCB system [18]. Thus, exposure to THC, especially during critical windows of brain development, has the potential to disrupt the tightly regulated system.

Parallel to the increase in adolescent cannabis use, the percentage of adolescents and young adults experiencing certain types of psychiatric disorders has risen in the United States over the past decade, despite the lack of increase in adults [19]. In human studies, THC has been shown to disrupt the development and function of the brain [20, 21], and in animals, THC has been experimentally shown to lead to molecular impairments that are heritable and extend into subsequent generations [22], thus increasing the risk of offspring developing a psychiatric disease [23–26]. Three different routes of multigenerational transmission have been summarized in a prior review [27]; they include fetal programming (direct effect), germline transmission (direct effect), and behavioral or social transfer (indirect effect). The first route is typical for prenatal exposure, the second route is typical for pre-gestational exposure, while the third route is typical for both.

In a recent commentary, which was published in response to a study examining the epigenetic impact of cannabis use on rat and human sperm [28••], the authors highlighted that the epigenomic toxicology of cannabinoids should have priority on the research agenda, especially considering the potential transgenerational health implications [29]. A review published in 2016 focused on the epigenetic effects of cannabis exposure [22]. The authors noted that the majority of addiction-related epigenetic neurobiological studies had targeted the adult brain, while there was a dearth of literature on the potential intergenerational impacts of cannabis [22]. Another article published in 2018 provided an overview of the current data regarding vulnerabilities of the developing brain to cannabinoid exposure during sensitive windows of development, especially with regard to epigenetic changes associated with cannabis use [27]. Since that time, additional studies were published that address research gaps and have the potential to better inform clinical guidelines, preventative policy, and public opinion related to cannabis use during specific time points of the life course.

Heritable molecular impairments include epigenetic modifications, such as DNA methylation, histone modifications, and changes in non-coding RNA (ncRNA), which regulate patterns of gene expression by altering DNA accessibility and chromatin structure [30]. DNA methylation occurs when a methyl group is added at a cytosine nucleotide that precede guanines (CpG dinucleotides), influencing DNA function by activating or repressing transcriptional activity of a gene and by altering chromatin accessibility and remodeling [30]. DNA methylation in the promoter region of a gene usually downregulates its expression, while higher DNA methylation in a gene body may promote expression of a gene. In most instances, DNA methylation represses gene expression by preventing the binding of transcription factors, or recruiting proteins that bind to methylated DNA [31]. Histones are large groups of protein complexes that help DNA condense into chromatin. Histone modifications include methylation and acetylation of lysine residues on histone tails, which affect gene expression by altering chromatin structure and accessibility [31]. In addition, ncRNA, such as micro (mi)RNA and long non-coding (lnc)RNA, control DNA availability and transcription, regulate RNA processing and splicing, and form a scaffold upon which layers of DNA regulation are built [29, 32].

Some epigenetic modifications are passed down to offspring through genomic imprinting (1% of mammalian genes), in which offspring only inherit one working copy of a gene. Imprinted genes are silenced via DNA methylation in either the egg or sperm [33]. Other modifications are passed down when genes escape epigenetic reprogramming, a process that occurs during the formation of primordial germ cells and in the early embryo soon after fertilization [34], in which genomic potential resets and epigenetic memory is erased [35].

In this review, we provide an analysis of the recent literature relating to pre-gestational and prenatal cannabinoid exposure and its effect on genes and molecular pathways. Along with the studies discussed in the review, additional animal studies are summarized in Tables 1 and 2, in which molecular changes are observed in the F0 generation of adolescent brain tissue [36–39, 40•, 41••, 42•].

**Table 1:** Summary of studies (from the past 10 years) on the effect of pre-gestational and prenatal cannabis exposure on genes and molecular pathways in humans. Studies are in reverse order chronologically by publication year

Reference	Study design	Time of exposure	Cell target	Outcome and platform	Main findings	Developmental significance
Schrott et al. 2019* [72••]	Cross-sectional study *n* = 24 male, adult subjects; 12 cannabis users and 12 controls	Pre-conception	Sperm of male adult subjects Human conceptal tissue (elective abortions) F0 generation	DNA methylation Quantitative bisulfite pyrosequencing mRNA transcript expression	• Methylation of *DLGAP2* intron 7 was inversely correlated with *DLGAP2* mRNA expression in human conceptal brain tissue (*p*-trend < 0.01). • The inverse relationship between *DLGAP2* gene methylation and mRNA transcript expression was evident for both males and females, but this relationship was significant only in females (*p*-trend = 0.006).	• *DLGAP2* is involved in synapse organization, neuronal signaling, and strongly implicated in autism spectrum disorder (ASD).
Gerra et al. 2018 [41••]	Nested case-control study *n* = 136 subjects ages 18–60 years; 40 cannabis users and 96 controls	Pre-conception	Peripheral whole blood of adult subjects F0 generation	DNA methylation Methylated DNA immunoprecipitation (MeDIP)-qPCR	• DNA methylation higher in cannabis users compared to control subjects in exon 8 of *DRD2* gene at + 66.7 kb from transcription start site (TSS) (*p* = 0.034) and CpG region at + 3 kb from TSS of *NCAM1* gene (*p* = 0.0004). • No difference in cannabis users compared to control subjects in DNA methylation at *ANKK1* − 0.25 kb, *DRD2* − 0.4 kb and + 0.9 kb, *NCAM1* + 0.4 kb, *CBR1* + 22.31 kb.	• *DRD2* and *NCAM1* play central role in dopaminergic pathway. • Increased methylation of *DRD2* and *NCAM1* may reflect lower mRNA expression. • Lower availability of *DRD2* receptors could underly reward deficit condition. • *NCAM1* implicated in developmental functions and nervous system maintenance.
Murphy et al. 2018* [28••]	Cross-sectional study *n* = 24 male, adult subjects; 12 cannabis users and 12 controls	Pre-conception	Sperm of male adult subjects F0 generation	DNA methylation Reduced representation bisulfite sequencing (RRBS)	• 6640 CpG sites differed (*p* < 0.05) between cannabis users and non-users. • Majority of CpGs (78.3%) had lower levels of methylation in user group. • *DGLAP2* hypomethylated in user group. • Maximum # CpG sites differentially methylated for given gene was for Aryl Hydrocarbon Receptor Repressor (AHRR) (94 CpGs hypomethylated ≥ 10% among users). • *PTG1R* methylation inversely correlated with Δ9-THC level (*R*^2^ = 0.839, *p* = 1.97e-4). • Increased *CSNK1E* methylation associated with increased Δ9-THC (*R*^2^ = 0.686, *p* = 0.003).	• DNA methylation changes of non-imprinted genes in gametes can resist post-fertilization reprogramming and persist in somatic cells of the offspring, including the brain. • *DGLAP2* gene encodes membrane associated protein involved in synapse organization and signaling in neuronal cells and is linked to schizophrenia. • *PTG1R*, which encodes the Prostaglandin I2 Receptor (a powerful vasodilator), associated with reduced sperm fecundity. • *CSNK1E*, which encodes the Casein Kinase 1 Epsilon, phosphorylates circadian clock protein PER2 and is implicated in sensitivity to opioids.
Fransquet et al. 2017 [62•]	Nested case-control study *n* = 804 maternal subjects; 44 cannabis users anytime during pregnancy and 760 non-user controls	Prenatal	Buccal cells of neonates of maternal subjects F1 generation	DNA methylation SEQUENOM MassARRAY	• Gestational cannabis use associated with increased methylation at one CpG site tested in *DRD4* (CpG.21.22.2) (β + 1.48, 95% CI: 0.02–2.93, *p* = 0.047). • At CpG.32, weak evidence that gestational cannabis is associated with increased methylation when adjusting for other substance use (β + .67, 95% CI: − 0.12-1.46, *p* = 0.09). • No associations remained significant after correction for multiple testing using a Bonferroni corrected significance level of 0.0026 given the 19 CpG units examined.	• *DRD4* plays key role in dopamine signaling and is associated with drug use. • Increased *DRD4* methylation in saliva has been associated with increased severity of ADHD symptoms in children. • Tissue specificity (using buccal cells instead of brain tissue) could have contributed to the null findings.
DiNieri et al. 2011* [55]	Case-control study *n* = 25 no maternal cannabis use and *n* = 24 maternal cannabis use	Prenatal	Fetal brain specimens (18–22 weeks of gestation) (elective abortions) F1 generation	Gene expression (mRNA)	• Decreased *DRD2* mRNA expression in NAc in brain specimens with maternal cannabis exposure (*p* = 0.003). • *DRD2* mRNA levels negatively correlated with maternal report of cannabis use (r = − 0.42, ***p*** **= 0.005**). • *DRD2* mRNA expression was not altered in putamen with cannabis exposure (*p* = 0.736). • *DRD1* mRNA levels were not altered in the NAc with cannabis exposure (*p* = 0.330). • *PDYN* mRNA expression was not altered with cannabis exposure (*p* = 0.155). • *PENK* mRNA expression was not altered with cannabis exposure.	• *DRD2* dysregulation implicated in addiction risk. • Cannabis could increase vulnerability to addiction and other psychiatric disorders by disrupting *DRD2* gene expression.

**Table 2:** Summary of studies (from the past 10 years) on the effect of pre-gestational, prenatal, and adolescent cannabis exposure on genes and molecular pathways in animals. Studies are in reverse order chronologically by publication year

Reference	Animal model	Type and time of exposure	Cell target	Outcome	Main findings	Developmental significance
Innocenzi et al. 2019 [15••]	Mice, male P7 CD-1; sexually mature	Synthetic cannabinoid receptor 2 (CBR2) agonist JWH-133 Pre-conception	Sperm F0 generation	DNA methylation MeDIP/hydroxymethylated DNA immunoprecipitation (hMeDIP)-qPCR	• Enrichment of 5mC at *Peg10* and *Plagl1* genes in sperm and placentas of exposed males, compared to controls. • No difference in *H19* methylation in sperm or placenta between groups. • Decreased *Tet3* mRNA expression in sperm of exposed males, compared to controls (*p* < 0.01), while decreased *Tet1* and *Tet2* gene expression was not significant. • No difference in *Dnmt* mRNA expression between groups. • Exposed males had decreased sperm count, impaired placental development, and reduced offspring growth, compared to controls.	• *H19* is maternally expressed imprinted gene that is a trans regulator of other imprinted genes during embryo growth. • *Peg10* is paternally expressed gene essential for placenta formation in humans and mice • *Plagl1* (*Zac*, *Lot1* and *Zac1* in mice) is paternally expressed and a key regulator of network of other imprinted genes, involved in embryonic growth and development.
Ibn Lahmar Andaloussi et al. 2019 [65•]	Rats, male Wistar; adolescence	Synthetic cannabinoid receptor 1 (CBR1) agonist WIN 55212–2 (WIN) Pre-conception F1 generation subjected to unpredictable or variable stress, or no stress	Brain tissue (prefrontal cortex [PFC]). F1 generation	DNA methylation 5-mc DNA Methylation ELISA kit	• Increased global DNA methylation in offspring with pre-conception WIN exposure, compared to controls (*p* < 0.05). • Increased DNA methyltransferase 1 (*Dnmt1*) mRNA level in offspring with WIN exposure, compared to controls (*p* < 0.01) in non-stressed animals. • Increased *Dnmt3a* mRNA expression in offspring with pre-conception WIN exposure, compared to controls (*p* < 0.05). • Correlation between global DNA methylation and *Dnmt1* expression (*p* = 0.035). • Stress exposure induced significant anxiety-like behavior in offspring with pre-conception WIN exposure, compared to controls (*p* < 0.001).	• DNA methyltransferase enzymes are essential for epigenetic maintenance. • Cocaine and alcohol exposure associated with dysregulated *Dnmt1* transcription in testes and sperm of adult male rodents (He et al. 2006 and Ouko et al. 2009).
Schrott et al. 2019* [72••]	Rats, male Sprague Dawley; sexually mature	Δ^9^-THC Pre-conception	Sperm F0 generation Brain tissue (hippocampus and nucleus accumbens [NAc])	DNA methylation Quantitative bisulfite pyrosequencing	• Using data from rats from this study (intravenous exposure) and Murphy et al. 2018 (oral exposure) identified region of discs-Large Associated Protein 2 (*Dlgap2*) with DM in eight CpG sites between exposed and control groups. • Sites 2, 3, 4 and 6 were significantly hypomethylated in exposed rats from the present study, compared to controls (*p*-trend = 0.03 to *p*-trend = 0.005). • In NAc, hypomethylation (*p*-trend = 0.02) at CpG site 2 in offspring (F1 generation), one of which was identified in the sperm of the Δ^9^-THC-exposed fathers.	• Intergenerational inheritance of altered DNA methylation in *Dlgap2* (despite evidence from the same study that it is not an imprint control region [ICR]). • *Dlgap2* strongly implicated in the development of autism spectrum disorder (ASD).
Miller et al. 2018 [40•]	Rats, male Long-Evans; adolescence	Δ^9^-THC Adolescence	Brain tissue (pyramidal neurons in layer III of prelimbic [PrL] subregion of rodent ventromedial PFC) F0 generation	mRNA transcript expression	• Δ^9^-THC associated with premature pruning of dendritic spines and allostatic atrophy of dendritic arborization in early adulthood. • Δ^9^-THC treated animals exhibited changes in genes associated with chromatin modification and histone methylation between adolescence and adulthood. • Enrichment analysis of differentially expressed genes showed strongest functional association with chromatin methyltransferase *Kmt2a* and histone H3 lysine 4 tri-methylation (H3K4me3).	• Pyramidal neurons comprise 80% of cortical neurons and express CB1R. • Disruption implicated in etiology of multiple psychiatric illnesses including schizophrenia. • *Kmt2a* mediates activity at *H3K4* and is highly implicated in cellular processes linked to neurodevelopment and psychiatric disorders.
Murphy et al. 2018* [28••]	Rats, male Sprague Dawley; sexually mature	Δ^9^-THC Pre-conception	Sperm F0 generation	DNA methylation Reduced representation bisulfite sequencing (RRBS)	• 627 genes had altered DNA methylation associated with Δ^9^-THC. • Topmost pathways with altered DNA methylation: ‘Hippo signaling pathway’ (3.6-fold enrichment; 17 genes, Bonferroni *p* = 0.004), and ‘Pathways in cancer’ (2.3-fold enrichment; 29 genes, Bonferroni *p* = 0.009), including six overlapping genes in each pathway from the human sperm (see human table). • Compared the 627 genes exhibiting differential DNA methylation in the rat sperm to the 473 differentially methylated genes identified from the brains of rat pups with pre-conception Δ^9^-THC exposure in Watson et al. 2015 (included in this table) and found 55 overlapping genes between the two datasets.	• DNA methylation changes of non-imprinted genes in gametes can resist post-fertilization reprogramming and persist in somatic cells of offspring, including the brain. • Alterations in the ‘Hippo signaling pathway’ and ‘Pathways of cancer’ could be retained in the zygote and disrupt expression of growth regulatory genes, potentially increasing cancer risk.
Tomas-Roig et al. 2017 [42•]	Mice, male C57Bl6/J; adolescence	WIN Adolescence	Brain tissue (hippocampus) F0 generation	DNA methylation Magnetic methylated DNA Immunoprecipitation (MagMeDIP) kit mRNA transcript expression	• The group treated with the highest level of WIN had DNA hypermethylation at the intragenic region of intracellular signaling modulator *Rgs7,* accompanied with a lower rate of mRNA transcription, compared to controls. • The group with the highest level of WIN exposure had increased N-arachidonoylethanolamine (AEA) levels, compared to controls. • Mice treated with WIN had memory impairment in Morris water maze, as well as dose-dependent memory impairment in fear conditioning.	• *Rgs7* plays key role in regulation of several biological processes such as vision, memory, motor control, reward behavior and nociception.
Hollins et al. 2016 [38]	Rats, Wistar; adolescent	HU210 (synthetic cannabinoid) Adolescence *A portion of the rats had maternal exposure to a viral mimic poly I:C, while others had maternal exposure to a vehicle	Brain tissue (left hemisphere of the entorhinal cortex [EC]) F0 Generation	mRNA-miRNA expression	• Gene expression alterations primarily in virus and cannabinoid group, with 195 genes showing differential expression (76% downregulated) compared to controls. • Cannabinoid only group had no genes unique to that group. • Functional annotation analyses revealed strong presence of genes involved in various aspects of cellular function, including Neurological Disease, Psychological Disorders, Cellular Development, Cellular Growth and Proliferation. • Comparison of mRNA expression data with previously described miRNA expression data (Hollins et al., 2014) revealed 82 miRNA-mRNA pairings, with nine unique miRNA targeting 56 unique mRNA.	• EC strongly associated with schizophrenia. • 56 unique mRNA subjected to pathway and ontology analysis which identified multiple individual genes associated with schizophrenia and processes implicated in the pathophysiology of the disorder.
Szutorisz et al. 2016 [23]	Rats, male Long-Evans; adolescence	Δ^9^-THC Pre-conception	Brain tissue (NAc and dorsal striatum) F1 generation	mRNA transcript expression	• In adolescence, interactions evident between treatment and sex for cannabinoid receptor 1 (*Cbr1*) (*p* = 0.04); glutamate ionotropic receptor NMDA type subunit 2A (*Grin1*) (*p* = 0.01); *Grin2b* (*p* = 0.01). • In the adult NAc, significant main effect of Δ^9^-THC for *Grin2a* (*p* = 0.02), with pre-gestational Δ^9^-THC leading to decreased mRNA expression. • In the adult dorsal striatum, decreased mRNA levels observed in offspring with pre-gestational Δ^9^-THC, including *Cnr1* (*p* = 0.04), *Grin1* (*p* = 0.003), *Grin2a* (*p* = 0.001), *Grin2b* (*p* = 0.01), *Gria1* (*p* = 0.008), *Gria2* (*p* = 0.001), discs large MAGUK scaffold protein 4 (*Dlg4*) (*p* = 0.02), and *Dlgap3* (*p* = 0.004). • Strength and pattern of NAc gene correlations similar between male and female adult offspring. • In contrast, gene correlations with pre-conception Δ^9^-THC were stronger in the dorsal striatum for females, consistent with the locomotor disturbances only experienced by females.	• Altered genes are relevant to neuropsychiatric disorders. • Striatal circuitry plays essential role in behaviors related to reward processing, motivation, emotion and motor activity.
Watson et al. 2015 [25]	Rats, male and female Long-Evans; adolescence	Δ^9^-THC Pre-conception	Brain tissue (NAc) F1 generation	DNA methylation Enhanced Reduced Representation Bisulfite Sequencing (ERRBS)	• 406 hypermethylated and 621 hypomethylated DMRs in exposed offspring, compared to controls, including 3758 CpGs (q < 0.01). • DMRs preferentially located in gene bodies and downstream of transcription start sites (TSS). • Top enrichments: cell membrane function, animal behavior, synaptic organization, receptor activity, including proteins localized to cellular components of neurons and synapses. • DMRs overlapped with genes encoding regulators of synaptic plasticity and transmission, including glutamate and kainate receptors. • Hypomethylated DMR in first coding exon of *Grin2a* consistent with mRNA transcript expression differences in Szutorisz et al. 2014 cohort. • 5 out of 10 DMR-associated genes in the *Dlg4* network (*Dlgap2*, *Kcna5*, *Begain*, *Grin2a*, and *Dlg4*) had differential mRNA expression between Δ^9^-THC and control groups (*P* < 0.05).	• NAc linked to addiction vulnerability, compulsive behaviors and reward sensitivity. • GluRs mediate synaptic plasticity and transmission, with impacts on addiction behavior (Szutorisz et al. 2014). • Results present evidence that germline Δ9-THC exposure leads to DNA methylation changes in gene loci related to synaptic plasticity and glutamatergic pathways, that resist post-fertilization reprogramming.
Hollins et al. 2014 [39]	Rats, Wistar; adolescence	HU210 (synthetic cannabinoid) Adolescence *Subset of rats received maternal exposure to a viral mimic poly I:C, while other subset given vehicle	Brain tissue (left and right hemisphere of the EC) F0 generation	miRNA expression	• Cannabinoid-exposure-only group had seven miRNAs with differential expression; miR-23a upregulated 2.85-fold with respect to control group. • High proportion of differentially expressed miRNAs structurally associated by genomic position to long arm of chromosome 6 (6q32) (associated with the syntenic human locus in schizophrenia), and predicted to regulate pathways involved in synaptic remodeling, learning and memory formation. • *Mef2d* (transcription factor that regulates miRNA expression) downregulated 7.6-fold following HU210 exposure alone; animals given HU210 only during adolescence had enrichment of predicted target genes in MAPkinase signaling pathway.	• EC strongly associated with schizophrenia. • High proportion of differentially expressed miRNAs structurally associated by genomic position to long arm of chromosome 6 (6q32), which is the syntenic human locus in schizophrenia and predicted to regulate pathways involved in synaptic remodeling, learning, and memory formation.
Szutorisz et al. 2014 [24]	Rats, male Long-Evans; adolescence and adulthood	Δ^9^-THC Pre-conception	Brain tissue (NAc and dorsal striatum) F1 generation	mRNA transcript expression Protein level and receptor binding	• Increase in mRNA expression of *Cbr1* and glutamate receptors in NAc (*p* = 0.03 for *Cbr1*; *p* = 0.009 for *Grin2a*; *p* = 0.04 for *Gria2*) in exposed rats, compared to controls at adolescent time point. • Decrease in mRNA expression in dorsal striatum (*p* = 0.03 for *Cbr1*; *p* = 0.009 for *Drd2*; *p* = 0.02 for *Grin1*; *p* = 0.005 for *Grin2a*; *p* = 0.03 for *Gria1*; *p* = 0.03 for *Gria2*) at adult time point. • No impairments in mRNA levels of same genes studied in medial PFC and orbito-frontal cortex, brain regions that have direct connectivity with the striatum and associated with addiction vulnerability. • In a separate group of F1 male offspring, protein level of GluN1 subunit (the product of *Grin1* mRNA) was decreased (*p* = 0.04); GluN2B (the product of *Grin2b* mRNA) was also decreased in association with parental Δ9-THC exposure (*p* = 0.02). • Long term-synaptic depression (LTD) was most prominent in the dorsal striatum, compared to the NAc, and the LTD in the former was most prominent and significantly larger with a main effect of parental treatment (*p* = 0.007) in male F1 offspring. No effect detected in NAc. • Parental Δ^9^-THC exposure associated with increased work effort to self-administer heroin, with enhanced stereotyped behaviors during period of acute heroin withdrawal.	• Abnormal mRNA levels in the NAc and later in the dorsal striatum mirror transition from reward-oriented to habitual, compulsive drug-taking that normally typifies progression from recreational drug use to addiction disorder. • The activity of medium spiny neurons in the striatum is regulated by glutamatergic input, which contributes to forms of synaptic plasticity such as LTD, which is strongly associated with habitual behaviors and reinforcement learning and relies on NMDA receptors and CBR1.
Vassoler et al. 2013 [68]	Rats, female Sprague-Dawley; adolescence	WIN Pre-conception Subset of F1 generation given morphine (vs. no morphine)	Brain tissue (NAc and paraventricular nucleus [PVN]) F1 generation	mRNA transcript expression	• Following morphine challenge, significantly higher levels of *Oprm1* (*p* = 0.016) in NAc of WIN-exposed animals, compared to controls. • On the day of challenge, morphine-pretreated WIN-exposed animals demonstrated a significantly enhanced response to morphine compared to morphine-pretreated controls.	• *Oprm1* gene encodes at least three opioid receptors in humans; important role in dependence to other drugs of abuse via modulation of dopamine system.
DiNieri et al. 2011* [55]	Rats, female pregnant Long-Evans	Δ^9^-THC Prenatal	Brain tissue (NAc) F1 generation (males only)	Histone modifications mRNA transcript expression	• Prenatal Δ^9^-THC exposure associated with increase in repressive 2meH3K9 mark between − 1.8 kb (69% increase vs control) and − 3 kb (83% increase vs control) upstream of the DRD2 TSS and decreased 3meH3K4 in the *Drd2* gene in the NAc at PND 62. • Reduced 3meH3K4 and decreased Pol II at the *Drd1* gene locus associated with prenatal Δ^9^-THC, despite lack of alteration of *Drd1* transcripts at PND 62. • *Drd2* mRNA expression decreased by 40% in the NAc (*p* = 0.001), but not the dorsal striatum (*p* = 0.37), at PND 2, in the exposed group relative to controls. *Drd2* mRNA expression decreased by 30% in the NAc (*p* = 0.001), but not the dorsal striatum (*p* = 0.32), at PND 62 in the exposed group relative to controls. • Decreased *Drd2* binding sites in the NAc (*p* = 0.05), as well as increased sensitivity to opiate reward in adulthood, at PND 62, in the prenatal Δ^9^-THC-exposed group.	• Data suggest that the association between prenatal Δ^9^-THC exposure and addiction vulnerability can be explained, at least in part, by Δ^9^-THC -induced alterations in the epigenetic regulation of the *Drd2* gene in the NAc. • *Drd2* dysregulation implicated in addiction risk. • Developmental regulation of 2meH3K9 important for appropriate tissue-specific expression of a variety of gene loci at promoters and regulatory regions.
Gleason et al. 2012 [36]	Mice, C57BL6; adolescence or early adulthood	WIN Adolescence	Brain tissue (hippocampus) F0 generation	Protein levels	• Lower glutamate receptor type 5 (mGluR5) in WIN-treated mice, compared to controls in adolescents. • Higher monoacylglycerol lipase (MGL) and fatty acid amide hydrolase (FAAH) in hippocampus of WIN-treated mice, compared to controls in adults. • Mice treated with WIN during adolescence showed long-lasting deficits in sensorimotor gating and hippocampal-dependent contextual learning in adulthood, compared to controls. • Mice treated with WIN during adolescence had deficits in prepulse inhibition and contextual learning, compared to controls.	• mGluR5 critically involved in fear conditioning. • Modulators of mGluR5 being examined as treatments for schizophrenia • Genes coding MGL and FAAH proteins are critically involved in eCB signaling.
Tomasiewicz et al. 2012 [37]	Rats, male Long-Evans; adolescence	Δ^9^-THC Adolescence	Brain tissue (NAc) F0 generation	DNA methylation Chromatin immunoprecipitation	• Decreased H3K9 methylation in Δ^9^-THC-exposed rats, compared to control rats, in the *Penk* gene.	• *Penk* opioid gene in the NAc competes with and mimics effects of opiate drugs, regulates heroin self-administration behavior, and plays role in pain, perception and stress response.

## Prenatal Exposure to Cannabis: Epigenetic and Functional Alterations in Offspring Brain Tissue

Since 2002, there has been an increase in pregnant women in the U.S. reporting daily cannabis use, use in the past-month, as well as an increase in the number of days during pregnancy that they report using cannabis [5]. Pregnant women report using cannabis most frequently during the first trimester [5], in order to mitigate morning sickness [43]. Studies have confirmed that THC readily crosses the placenta, distributes into the fetal compartment, and crosses the fetal blood-brain barrier [44]. A handful of studies in both human subjects and animal models have indicated that the embryonic nervous system patterning is particularly susceptible to maternal cannabis use [45–50]. Its use during pregnancy has been associated with an increased risk of various cognitive, behavioral, and neuropsychiatric defects [51, 52]. Use during pregnancy has also been associated with an increased risk of preterm birth in some studies [49, 50], as well as decreased birth weight [53, 54]. This section highlights recent studies that have examined the epigenetic mechanisms by which prenatal cannabis exposure increases the risk of postnatal psychiatric disease.

### Human Evidence

Considering that maternal cannabis use during pregnancy is associated with long-term adverse behavioral outcomes and addiction vulnerability in offspring [44], it is possible that epigenetic changes established in utero that affect dopaminergic reward signaling are involved. The striatal dopamine system, composed of medium spiny neurons enriched in cannabinoid receptors, is implicated in the pathogenesis of neuropsychiatric disorders [55]. One study tested the neurobiology underlying the risk of addiction vulnerability in humans by examining mRNA expression in fetal brain specimens of the putamen and nucleus accumbens (NAc), from mothers who underwent elective abortions between 18 and 22 weeks of gestation [55] (Table 1). Half of the fetal brain specimens were those from mothers who had positive maternal self-report and/or maternal urine that tested positive for THC and/or fetal meconium positive for THC, while the other half had no cannabis exposure. Not only did fetuses exposed prenatally to cannabis have decreased dopamine receptor D2 (*DRD2*) mRNA levels in the NAc, compared to controls, but there was also a dose response observed in which greater maternal use was correlated with decreased *DRD2* mRNA levels. In contrast, there was no difference in *DRD2* mRNA levels in the putamen. There was also no difference in *DRD1* mRNA levels, or mRNA levels of the opioid neuropeptides proenkephalin (*PENK*) and prodynorphin (*PDYN*) in the putamen or NAc, between the exposed and unexposed groups. The NAc core and shell are important components of motor and reward circuits, respectively [56], and disruptions in these signaling pathways could lead to adverse psychiatric outcomes [57].

Additional studies were conducted on the same fetal brain specimens used in the study discussed above [55]. In these analyses, decreased *DRD2* mRNA levels were observed in the amygdala basal nucleus of fetuses exposed prenatally to cannabis compared to controls [58], which was consistent with the reduced levels observed in the NAc [55]. In addition, fetal brain specimens with maternal cannabis exposure had reduced *PENK* expression in the caudal putamen, and *PENK* mRNA levels were inversely correlated with amount of maternal cannabis intake during pregnancy [59]. Disruptions in the opioid system during development contribute to the development of psychiatric disorders [59, 60] and persist into adulthood, increasing vulnerability to opiate-seeking behavior [61]. Dysregulation of *DRD2* is implicated in addiction risk and other psychiatric disorders, and its alteration was a consistent finding in the animal studies, as well as the human studies.

Another recent study evaluated whether prenatal cannabis exposure is associated with DNA methylation of dopamine receptor D4 (*DRD4*) promoter in buccal cells from the neonates of maternal subjects with either cannabis or no cannabis use anytime during pregnancy [62•] (Table 1). Buccal epithelial cells have the same developmental origins as neuronal cells, and prior studies provide support for buccal cells as a proxy for neurodevelopmental phenotypes [63]. There was no association between DNA methylation at individual CpG sites in *DRD4* after correction for multiple testing. It is unclear if the null findings were due to the relatively small sample size (*n* = 804 maternal subjects), the tissue specificity, or a lack of biological relevance [62•]. Certain genetic polymorphisms of *DRD4* increase risk of drug use and severity of ADHD symptoms in children, both of which have been associated with cannabis exposure [64]. Future candidate gene studies should examine the association between prenatal cannabis exposure and epigenetic changes in *DRD4* in brain or other target cells, instead of the buccal cell proxy, as well as account for genetic polymorphisms.

### Animal Evidence

The study discussed above [55] that reported decreased *DRD2* mRNA levels in the NAc of cannabis-exposed gestation week 18–22 human fetuses also explored the mechanisms underlying this decrease in studies in rats (Table 2). Pregnant rats were exposed to THC and changes in NAc *Drd2* gene expression in offspring were evaluated at postnatal day 2 (PND2), a comparable point in brain development as that occurring in humans during gestation weeks 18–22 [55, 58, 59]. A separate cohort of male offspring was studied in adulthood (PND62), in order to evaluate any long-term impairments induced by prenatal THC exposure. Consistent with the cannabis-exposed human fetuses, rats exposed to THC had about a 40% reduction in NAc *Drd2* mRNA levels at PND2, compared to unexposed control rats. This decrease persisted into adulthood, with about a 30% reduction in NAc *Drd2* mRNA levels observed at PND62 in prenatally exposed rats, compared to unexposed controls. There was no difference in *Drd2* mRNA levels, between the exposed and unexposed groups, in the dorsal striatum [55].

In this study [55], the epigenetic mechanisms by which *Drd2* mRNA transcript expression was altered were evaluated. THC exposure significantly increased the repressive dimethylated lysine 9 (2meH3K9) mark on histone H3 between − 1.8 kb (69% increase vs control) and − 3 kb (83% increase vs control) upstream of the transcription start site (TSS) in the *Drd2* gene. It also decreased trimethylated lysine 4 (3meH3K4) on histone H3 across the analyzed genomic fragment in the NAc of adult rats. Consistent with 3meH3K4 acting as a mark of transcriptional activity, its reduction was associated with decreased RNA polymerase II (Pol II) at the TSS (+ 0.3 kb) and within the coding region(+ 40 kb). Although no change in 2meH3K9 was observed at the dopamine receptor D1 (*Drd1*) gene, there was reduced 3meH3K4 and decreased Pol II association at this locus, despite the lack of alteration of *Drd1* transcripts in the NAc during adulthood. Decreased dopamine receptor binding sites were also observed in the adult NAc in the THC-exposed rats, compared to controls.

## Pre-gestational Exposure to Cannabis: Epigenetic and Functional Alterations in Offspring Brain Tissue

There is some evidence in model animal studies that pre-gestational exposure to cannabis also causes alterations that can be passed down to offspring, even after years of cannabis cessation. It is possible that epigenetic modifications mediate the relationship between pre-gestational exposure to cannabis and adverse psychiatric outcomes in offspring, especially when cannabis exposure occurs during adolescence or early adulthood.

### Animal Evidence

A recent study evaluated the association between male rat exposure to synthetic CBR1 agonist WIN 55212-2 (WIN) during adolescence (compared to pre-gestational vehicle [VEH] exposure) and global DNA methylation in the prefrontal cortex (PFC) of their offspring. The offspring were also subjected to unpredictable stress, variable stress, or no stress, in order to examine the interaction between pre-gestational WIN exposure and stress response [65•] (Table 2). Increased global DNA methylation was observed in offspring with pre-gestational WIN exposure, compared to controls, regardless of presence or absence of stress exposure. In addition, increased DNA methyltransferase (*Dnmt*)*1* mRNA levels were observed in offspring with pre-gestational WIN exposure, compared to unexposed controls in non-stressed animals only, while increased *Dnmt3* mRNA levels were observed in offspring with pre-gestational WIN exposure, compared to unexposed controls, regardless of presence or absence of stress exposure. It is plausible that the increased global PFC DNA methylation observed in animals with pre-gestational WIN exposure, as well as in stressed animals, was mediated by the upregulation of DNMT enzymes, since these are responsible for epigenetic maintenance. The molecular alterations were consistent with the observed phenotypes, as stress exposure induced anxiety-like behavior in offspring with pre-gestational WIN exposure, compared to controls without pre-gestational WIN exposure. The epigenetic changes in the offspring could have been due to direct epigenetic modifications on the sperm or testes, as well as related to disruptions in the paternal endocannabinoid system. This animal model supports a transgenerational epigenetic effect of cannabinoid exposure potentially altering stress response in the offspring. However, global DNA methylation measurements lack gene specificity and therefore provide limited biological insights.

Another study in rats examined the association between exposure to THC in male and female rats during adolescence and differentially methylated regions (DMRs) in the NAc of offspring using Enhanced Reduced Representation Bisulfite Sequencing (ERRBS) [25] (Table 2). A total of 1027 DMRs, including 406 hypermethylated and 621 hypomethylated regions, were observed in exposed offspring, compared to unexposed controls with genes enriched for cell membrane function, synaptic organization, and receptor activity. The hypomethylated DMR in the first coding exon of glutamate ionotropic receptor NMDA type subunit 2A (*Grin2a*) was consistent with *Grin2a* mRNA transcript expression differences observed in another rat study by the same research group [24] (Table 2). This is in line with the hypothesis that hypomethylation in gene bodies may lead to decreased gene expression [66]. The *Grin2a* gene is involved in calcium channel activity and ionotropic glutamate receptor activity and mediates synaptic plasticity and transmission, with impacts on addictive behavior [24, 67].

The same authors that observed reduced *Grin2a* mRNA levels in the NAc also observed differential mRNA gene expression in different areas of the brain, depending on the time of evaluation (adolescence versus adulthood) in rats pre-gestationally exposed to THC [24] (Table 2). There was increased *Cbr1*, *Grin2a*, and *Gria2* mRNA expression in pre-gestationally exposed rats, compared to controls at the adolescent time point, while there was a decrease in mRNA expression of *Cbr1*, *Drd2*, *Grin1*, *Grin2a*, *Gria1*, and *Gria2* in the dorsal striatum in pre-gestationally exposed rats, compared to controls, at the adult time point. The shift in mRNA expression from the adolescent to adult time point is consistent with the transition from reward-oriented to habitual, compulsive drug-taking that typifies progression from recreational drug use to addiction disorder. The same study authors further evaluated potential sex-specific effects and observed that sex-specific mRNA expression patterns were present in both the adolescent and adult brains [23] (Table 2). Overall, the findings contribute to evidence that parental history of germline THC exposure could possibly confer enhanced risk for psychiatric disorders in the subsequent generation, as a result of impaired epigenetic regulatory processes in relevant genes and pathways.

One other study observed no changes in *Drd2* mRNA levels in the NAc of adult rats exposed pre-gestationally to WIN and postnatally to morphine [68] (Table 2). The latter study, however, found higher opioid receptor mu (*Oprm*)*1* mRNA levels in WIN-exposed animals, compared to unexposed animals, following a morphine challenge. On the day of the morphine challenge, animals pre-gestationally exposed to WIN had an enhanced response to morphine, compared to controls. The *Oprm1* gene encodes at least three opioid receptors in humans, and it is involved in dependence to drugs such as nicotine, cocaine, and alcohol via its modulation of the dopamine system. The study contributed to evidence that pre-gestational cannabis could induce addiction vulnerability in F1 offspring.

## Pre-gestational Exposure to Cannabis: Epigenetic and Functional Alterations in Parental Sperm

Not only are epigenetic and functional alterations observed in brain tissue of offspring with pre-gestational exposure to cannabis, but there is also evidence of epigenetic and functional alterations in sperm of exposed individuals in the F0 generation, which could promote germline epigenetic inheritance in future generations.

### Human Evidence

In order to evaluate the impact of cannabis exposure during adulthood on the sperm methylome, one study examined DNA methylation in adult male subjects that had either cannabis or no cannabis use [28••] (Table 1). Over 6000 CpG sites differed between cannabis and non-cannabis users. Specifically, prostaglandin I2 receptor (*PTGIR*) methylation was inversely correlated with THC level, while casein kinase 1 epsilon (*CSNK1E*) methylation was associated with increased THC. The *PTGIR* gene is associated with reduced sperm fecundity, while *CSNK1E* phosphorylates period circadian regulator (*PER*)*2* and is implicated in sensitivity to opioids [67]. Discs-Large Associated Protein (*DGLAP*)*2* was also hypomethylated in the sperm of cannabis-exposed men, compared to controls [28••]. The *DGLAP2* gene encodes a membrane-associated protein that is involved in synapse organization and signaling in neuronal cells [67] and is linked to psychological and neurological disorders, such as schizophrenia [69]. It has also been identified as an autism candidate gene [70, 71]. While it is biallelically expressed in the brain, only the paternal allele is expressed in the testes due to imprinting [67].

Another recent study by Schrott et al. 2019 [72••] further evaluated DNA methylation and mRNA transcript expression using the same study population as the study discussed above [28••] (Table 1). They first validated the findings related to *DLGAP2* in the study discussed above using quantitative bisulfite pyrosequencing, instead of reduced representation bisulfite sequencing (RRBS) [28••], which showed good agreement. The authors noted that it was one of 46 genes with more than 10 CpG sites showing altered DNA methylation in the sperm of cannabis users, compared to controls. They observed sperm hypomethylation of *DLGAP2* at 17 CpGs in exposed adult men, compared to controls, in the RRBS assay. The differential DNA methylation was validated in *DLGAP2* for nine CpG sites, plus an additional site, in intron 7 in the pyrosequencing assay. They further determined the functional association between DNA methylation and mRNA transcript expression in human brain tissue from terminated pregnancies, a relevant target tissue for the expression of *DLGAP2*. In these human brain samples, methylation of *DLGAP2* intron 7 was inversely correlated with *DLGAP2* mRNA expression and significant only in females.

### Animal Evidence

The same two studies in human sperm highlighted in the preceding section also validated their findings using male, sexually mature adult rat sperm from animals exposed to THC, compared to unexposed controls. In the first study, 627 genes had altered DNA methylation associated with THC exposure [28••] (Table 2). There were six overlapping genes among the rat and human-exposed sperm, suggesting that these two pathways may be targets of THC exposure across species. Although the study focused on the F0 generation, some DNA methylation changes of non-imprinted genes in gametes can resist post-fertilization reprogramming and persist in the somatic cells of offspring [73]. Supporting this hypothesis, the authors compared the 627 genes exhibiting DMRs in the rat sperm [28••] to the 473 DMR genes identified in the NAc of adult rats exposed to THC pre-gestationally (compared to unexposed controls) in a study discussed earlier in this review [25] (Table 2). They found 55 overlapping DMR genes between these two datasets with significant enrichment, suggesting that THC-induced epigenetic modifications in sperm cells could persist in somatic cells. Important strengths of this study include some validation from similar observations in human sperm and in adult rat brain tissue.

The same authors pooled data from a new set of sexually mature rats that were given intravenous THC, with the set of rats from Murphy et al. 2018 [28••] given oral THC, and identified a region of *Dlgap2* that showed differential methylation in eight CpG sites in sperm between exposed and control groups. Hypomethylation at CpG site 2 was detected in the NAc of pre-gestationally exposed offspring (F1 generation), compared to controls, as well as in the sperm of the THC exposed fathers, compared to controls [72••] (Table 2). The study provided evidence of potential intergenerational inheritance of epigenetic marks in *Dlgap2*, despite evidence from the same study that it is not an imprint control region.

Finally, a recent study examined epigenetic and functional alterations in sperm of sexually mature mice exposed to the synthetic CBR2 agonist JWH-133. Not only did exposed males have decreased sperm count, but their offspring demonstrated impaired placental development and reduced growth, compared to unexposed controls. This was accompanied by increased DNA methylation at the paternally expressed imprinted genes *Peg 10* and *Plagl1* in sperm, which was retained in the offspring placenta [15••]. Although the study highlights that overactivation of CBR2 can promote altered DNA methylation in sperm, which is retained in embryonic tissue and may cause altered offspring phenotypes, it could not confirm the precise effect that the epigenetic alterations may have on offspring. However, considering that cannabis is made up of numerous cannabinoids that could bind with CBR2, it adds to the body of evidence that pre-gestational cannabis may promote epigenetic changes in sperm cells that are functionally relevant in offspring.

## Conclusion

Together, these findings suggest that pre-gestational and developmental cannabis exposure alters epigenetic processes like DNA methylation and histone modifications with functional consequences for gene expression. Fetal epigenetic programming of genes and some molecular pathways are suggestive of alterations in regions involved in the development of autism spectrum disorder (ASD), attention deficit hyperactivity disorder (ADHD), schizophrenia, addiction, and other psychiatric diseases.

The overall body of evidence is of high public health relevance, as attitudes about cannabis use are changing in favor of its use, especially in adolescents and young adults. In the 2018 National Survey on Drug Use and Health, the majority of people aged 12 or older perceived great risk of harm from weekly use of cocaine or heroin (86.5% and 95.3%, respectively), while less than one third of people (30.6%) aged 12 or older perceived great risk of harm from weekly cannabis use [74]. The legal cannabis market is capitalizing on its popularity by implementing selective growing methods to boost psychoactive potency and to increase profits [75]. In fact, over the last two decades, the average THC content of cannabis has increased from four to 12% [75]. Levels as high as 30% have recently been documented in legal cannabis grown for recreational use [76]. Additionally, ease of delivery methods such as cannabis vaping can increase its reach to younger groups.

According to the 2018 National Survey on Drug Use and Health, more than a third of young adults aged 18 to 25 (34.8%) were past year users of cannabis, or about 11.8 million young adults. A lower percentage of adults ages 26 years or older reported using cannabis in the past year (13.3%), compared to the young adult age group. However, the percentage of adults reporting use of cannabis in the past year was higher than had been reported in surveys conducted between 2002 and 2016. About 3.1 million people aged 12 or older used cannabis for the first time in the past 12 months, translating to about 8400 new cannabis users each day [74].

The main gaps in the literature are the lack of human studies on pre-gestational exposure to cannabis, as well as the lack of studies examining the transgenerational effect of cannabis exposure. Limitations in the body of literature examined in this review include limited statistical power from low sample sizes, limitations in exposure quantification in human studies, and differences in dosage, timing of exposure, and tissue and cell types analyzed for epigenetic endpoints. Human exposure to cannabis is complex due to different delivery methods, THC/CBD ratios, and timing of exposure during critical developmental periods (e.g., adolescence, pre-gestational, and prenatal). However, studies that incorporated mixed study designs that examined the effects of developmental cannabis exposure on both animals and humans, or that compared findings to other studies, found consistent epigenetic and functional gene changes between species and between studies [23–25, 28••, 55, 72••], which is rare in epigenetic studies. In addition, significant effects were observed in cannabis-exposed subjects in the majority of studies, despite low sample sizes. Yet, publication bias could be an important driver. The field would benefit from a large meta-analysis to increase power, particularly from human studies to uncover novel epigenetic biomarkers. The majority of experimental and epidemiological studies have examined differences in DNA methylation. Future studies should also incorporate the measurement of histone modifications, changes in ncRNA, and persistence over time in sperm, which could yield valuable information in transgenerational studies [77].

Cannabis consumption and mental health illness in young adults is increasing in the United States. Developmental cannabinoid exposure may increase the intergenerational risk of psychiatric disease through epigenetic mechanisms. These findings could be used by regulators and health communicators to inform consumers of potential risks associated with cannabis use during specific time points in the life course.
